# The association between fear of progression and medical coping strategies among people living with HIV: a cross-sectional study

**DOI:** 10.1186/s12889-024-17969-1

**Published:** 2024-02-12

**Authors:** Bing Li, Xiaoli Lin, Suling Chen, Zhe Qian, Houji Wu, Guichan Liao, Hongjie Chen, Zixin Kang, Jie Peng, Guangyu Liang

**Affiliations:** Department of Infectious Diseases, Nanfang Hospital, Southern Medical University, Guangzhou, 510515 China

**Keywords:** Fear of progression, HIV infection, Metal health, Internalized HIV stigma, Medical coping modes, Social support

## Abstract

**Background:**

Due to the chronic nature of HIV, mental health has become a critical concern in people living with HIV (PLWHIV). However, little knowledge exists about the association between fear of progression (FoP) and medical coping modes (MCMs) in PLWHIV in China.

**Methods:**

A cohort of 303 PLWHIV were consecutively enrolled and their demographic, clinical and psychological information was collected. The Fear of Progression Questionnaire-Short Form (FoP-Q-SF), Social Support Rating Scale (SSRS), Internalized HIV Stigma Scale (IHSS) and MCMs Questionnaire were utilized.

**Results:**

Of the participants, 215 PLWHIV were classified into the low-level FoP group, and 88 were grouped into the high-level FoP group based on their FoP-Q-SF scores, according to the criteria for the classification of dysfunctional FoP in cancer patients. The high-level group had a higher proportion of acquired immunodeficiency syndrome (AIDS) stage (*P* = 0.005), lower education levels (*P* = 0.027) and lower income levels (*P* = 0.031). Additionally, the high-level group had lower scores in social support (*P* < 0.001) and its three dimensions, with total SSRS scores showing a negative correlation with two dimensions of FoP-Q-SF, namely physical health (r^2^ = 0.0409, *P* < 0.001) and social family (r^2^ = 0.0422, *P* < 0.001). Further, the high-level group had higher scores in four dimensions of internalized HIV stigma, and a positive relationship was found to exist between IHSS scores and FoP-Q-SF scores for physical health (r^2^ = 0.0960, *P* < 0.001) and social family (r^2^ = 0.0719, *P* < 0.001). Social support (OR = 0.929, *P* = 0.001), being at the AIDS stage (OR = 3.795, *P* = 0.001), and internalized HIV stigma (OR = 1.028, *P* < 0.001) were independent factors for FoP. Furthermore, intended MCMs were evaluated. FoP were positively correlated with avoidance scores (r^2^ = 0.0886, *P* < 0.001) and was validated as the only factor for the mode of confrontation (OR = 0.944, *P* = 0.001) and avoidance (OR = 1.059, *P* = 0.001) in multivariate analysis.

**Conclusion:**

The incidence of dysfunctional FoP in our study population was relatively high. High-level FoP was associated with poor social support, high-level internalized HIV stigma and a negative MCM among PLWHIV.

## Background

Since the clinical advent of highly active antiretroviral therapy (HAART), the life expectancy of people living with HIV (PLWHIV) has significantly increased [1]. Due to the chronic nature of HIV, their mental health has become a critical concern.

PLWHIV often experience psychological conditions that are directly linked to HIV infection, such as internalized HIV stigma (IHS). IHS refers to negative self-beliefs associated with HIV and contributes to poor treatment adherence and unsuppressed HIV viral load [2–4]. In contrast, social support has been widely studied for its protective role in mental health and is associated with good treatment adherence and undetected HIV RNA among PLWHIV [5, 6].

Fear of progression (FoP) is a type of anxiety that results from concerns about disease progression and its consequences [7]. FoP is a significant mental health issue that has been studied first among diseases such as cancer, diabetes mellitus and rheumatic diseases [7]. Previous research has shown that FoP levels differ depending on the tumor stage among cancer patients [8, 9]. However, to date, no studies have investigated the level of FoP among PLWHIV at different stages of HIV infection.

FoP level has been found to be associated with their coping orientation among breast cancer survivors [10]. Medical coping modes (MCMs) describes the different coping strategies used by patients to manage their disease, including three modes: confrontation, avoidance, and resignation [11]. Coping styles can vary among patients with different diseases [12]. Among PLWHIV, emotional coping strategies are commonly employed, with confrontation being the most frequently used coping style [13, 14]. Previous research has demonstrated that coping styles can have a significant impact on patient outcomes. For example, patients with advanced gastric cancer who adopted the confrontation mode had higher quality of life (QoL) scores, while those who used the avoidance or resignation mode had lower QoL scores [15].

Similar to other chronic diseases, HIV infection has different stages of progression, consisting of acute HIV infection, chronic HIV infection, and acquired immunodeficiency syndrome (AIDS) stage. Without positive treatment willing, PLWHIV may eventually progress to the AIDS stage, which is characterized by development of opportunistic infections and tumours [16–18]. HAART significantly reduces HIV replication and delays disease progression, making adherence to medication schedules crucial [19, 20]. However, previous research has demonstrated that negative coping modes can harm PLWHIV treatment adherence, thereby significantly impairing the effectiveness of anti-HIV treatment [21, 22].

This research utilized the Fear of Progression Questionnaire-Short Form (FoP-Q-SF) to assess FoP levels among PLWHIV. Based on the abovementioned findings, we hypothesized that PLWHIV in different HIV infection stages might exhibit different FoP levels, which could be correlated with different MCMs. Early identification of PLWHIV with high FoP levels and appropriate interventions might help mitigate negative consequences. Therefore, we conducted a cross-sectional study to assess FoP levels and related factors among PLWHIV, as well as to investigate the potential impact of FoP on MCMs.

## Methods

### Study population and design

The study was a cross-sectional study. The minimum sample size was calculated and 303 participants were selected using a random sampling method from approximately 2000 PLWHIV, who were routinely monitored and followed up at the Department of Infectious Diseases, Nanfang Hospital of Southern Medical University. Informed consent was obtained from all participants before inclusion in the study. All enrolled individuals completed several questionnaires, including the FoP-Q-SF, Social Support Rating Scale (SSRS), Internalized HIV Stigma Scale (IHSS) and Medical Coping Modes Questionnaire (MCMQ), in a quiet room without any distractions or interruptions. Participants were instructed to seek professional help if they had any difficulty in understanding the questionnaires.

### Demographic parameters and laboratory tests

Baseline characteristic data of all enrolled PLWHIV were collected, including age, sex, being married, having children, education level and income level. Education level was divided into low (≤ 12 years) and high (> 12 years), and income level was divided into low (per capita annual household income ≤ 60,000 yuan) and high (per capita annual household income > 60,000 yuan) according to per capita disposable income in Guangzhou City [23]. Laboratory tests included HIV viral load, CD4 + T-cell counts, and CD8 + T-cell counts. HIV viral load was detected by polymerase chain reaction, and considered undetectable when viral RNA was below the lower limit of detection (100 copies/ml). CD4+, CD8 + T-cell counts were determined by flow cytometry. All tests and evaluations were conducted according to the manufacturer’s instructions at the Department of Clinical Laboratory of Nanfang Hospital.

### Psychological measurements

#### Fear of progression questionnaire-short form

The FoP-Q-SF is used to assess FoP, which consists of 12 items covering two dimensions: physical health and social family. Each item is scored using a 5-point Likert scale ranging from 1 (never) to 5 (regular). The total score ranges from 12 to 60. A higher score indicates greater fear of disease progression. A score of 34 or above indicated a dysfunctional level of FoP and was the cutoff value for the high-level FoP group in this research [24]. In this study, we applied the Mandarin version of the FoP-Q-SF, and the Cronbach’s alpha was 0.91 in this sample.

#### Social support rating scale

The SSRS includes 10 items divided into three dimensions: objective support, subjective support and utilization of support. Items 1–4 and 8–10 are rated on a 4-point Likert scale ranging from 1 (not at all) to 4 (very much), while item 5 includes five parts, each rated on the same 4-point scale. For Items 6 and 7, if “No source” is answered, a score of 0 is given; if “have a source” is answered, each source is given 1 point. The total SSRS score ranges from 12 to 66, with higher scores indicating better social support. Cronbach’s alpha of SSRS was 0.66 in this study.

#### Internalized HIV stigma scale

IHSS is a 28-item scale designed to assess negative self-beliefs associated with HIV. It includes four dimensions: stereotypes, disclosure concerns, social relationships, and self-acceptance. Responses were recorded on a 4-point Likert scale, ranging from 0 (never) to 4 (all the time). Higher scores on the IHSS indicate greater levels of internalized stigma [25]. Cronbach’s alpha of IHSS was 0.95 in this study.

#### Medical coping modes questionnaire

The MCMQ is used to assess the coping strategies of individuals who have undergone medical events. The MCMQ is composed of 20 items, which are classified into three dimensions: confrontation, avoidance, and resignation. Each item is rated on a 4-point scale ranging from 1 to 4, with eight items being graded in reverse. A higher cumulative score for each dimension indicates a higher likelihood of individuals to adopt the corresponding coping mode. The Chinese version of the MCMQ has good internal reliability [26]. Cronbach’s alpha of the three dimensions were 0.75, 0.68 and 0.79, respectively.

### Illustration tool

The illustration in conclusion was created with BioRender (https://biorender.com).

### Statistical analysis

In this study, we performed a power calculation using G*Power 3.1.9.7 (https://g-power.apponic.com/). For 90% power with α set at 0.05, a total sample size of 86 participants was needed. Continuous variables and categorical variables were reported as the mean ± standard deviation and frequency with percentage respectively. To determine the differences in results, chi-square tests or t tests were used. Linear correlation analysis was performed to determine the correlation between FoP and social support and IHS. Logistic regression analysis was used to analyse associated factors. The significance level was set as *P* < 0.05 (two-tailed). Statistical analysis was performed using SPSS software (Version 26.0). Figures were generated in GraphPad Prism 9.0, R Version 4.2.2 and Adobe Illustrator CC2020.

## Results

### Baseline characteristics of PLWHIV enrolled

A total of 303 PLWHIV were included in the study. Among all PLWHIV, 215 (71.0%) individuals were grouped into the low-level FoP group, and 88 (29.0%) individuals were included in the high-level FoP group. Their baseline characteristics are shown in Table 1. There were no significant differences in age, sex, HIV viral load, CD4 + T-cell counts, CD8 + T-cell counts, being married or having children between the two groups. The high-level group had a higher proportion of AIDS stage (20.45% versus 7.50%, *P* = 0.005), lower education (62.50% versus 48.60%, *P* = 0.027) and lower income levels (69.32% versus 56.28%, *P* = 0.031).

**Table 1:** Demographics and clinical characteristics in PLWHIV enrolled

Variable	FoP Scores of PLWHIV		
	Low-Level Group	High-Level Group	***P*** value
**Age (years)**	31.94 ± 20.53	30.83 ± 8.02	0.623
**Sex**			
**Male**	212 (98.60)	83 (94.32)	0.103
**Female**	3 (1.40)	5 (5.68)	
**HIV Viral Load**			0.799
**Undetectable**	209 (97.21)	86 (97.73)	
**Detectable**	6 (2.79)	2 (2.27)	
**CD4 + T-cell Counts**	489.06 ± 215.17	451.11 ± 228.18	0.172
**CD8 + T-cell Counts**	799.08 ± 307.76	828.50 ± 353.69	0.471
**AIDS stage**			0.005*
**Yes**	15 (7.50)	18 (20.45)	
**No**	200 (92.50)	70 (79.55)	
**Being Married**			0.887
**Yes**	28 (13.02)	12 (13.64)	
**No**	187 (86.98)	76 (86.36)	
**Having Children**			0.277
**Yes**	37 (17.21)	20 (22.73)	
**No**	177 (82.79)	67 (77.27)	
**Education**			0.027*
**≤ 12 years**	104 (48.60)	55 (62.50)	
**> 12 years**	110 (51.40)	33 (37.50)	
**Income** ^**a**^			0.031*
**Low**	121 (56.28)	61 (69.32)	
**High**	94 (43.72)	27 (30.68)	

Abbreviations: AIDS: Acquired Immunodeficiency Syndrome; FoP: Fear of Progression; PLWHIV: People Living with HIV.

^a^Low: per capita annual income ≤ 60,000 yuan; High: per capita annual income > 60,000 yuan

*P value is less than 0.05

### Relationship between social support and fear of progression in PLWHIV

**Fig. 1:**
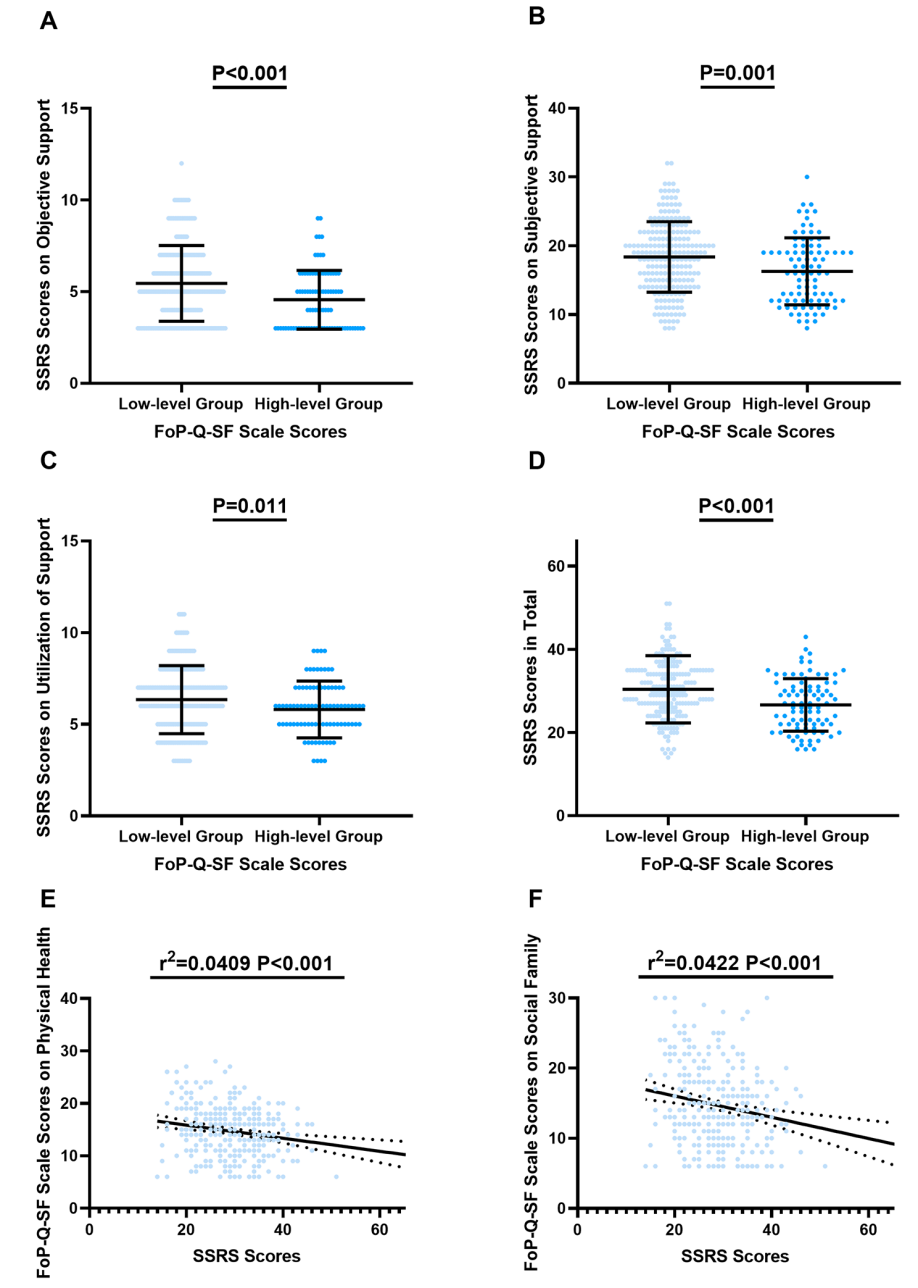
Relationship between FoP and SSRS The comparison of SSRS scores including objective support **(A)**, subjective support **(B)**, utilization of support **(C)** and total SSRS score **(D)** between groups characterized by low-level and high-level FoP. **(E)** The relationship between FoP-Q-SF scale scores on physical health and SSRS scores among PLWHIV. **(F)** The relationship between FoP-Q-SF scores on social family and SSRS scores among PLWHIV. Abbreviations: FoP: Fear of Progression; FoP-Q-SF: Fear of Progression Questionnaire-Short Form; PLWHIV: People Living with HIV; SSRS: Social Support Rating Scale

To analyse the correlation between social support and FoP, we compared the scores of the three dimensions of the SSRS between the high-level FoP group and the low-level FoP group. Compared with the low-level group, the high-level group had lower scores in three dimensions, including objective support (4.56 ± 1.60 versus 5.45 ± 2.07, *P* < 0.001) (Fig. 1A), subjective support (16.27 ± 4.87 versus 18.37 ± 5.13, *P* = 0.001) (Fig. 1B) and utilization of support (5.81 ± 1.55 versus 6.34 ± 1.85, *P* = 0.011) (Fig. 1C). The total SSRS scores were lower in the high-level group than in the low-level group (26.64 ± 6.31 versus 30.40 ± 8.07, *P* < 0.001) (Fig. 1D). Correlation analysis showed that social support was negatively correlated with FoP-Q-SF on physical health (r^2^ = 0.0409, *P* < 0.001) (Fig. 1E) and social family (r^2^ = 0.0422, *P* < 0.001) (Fig. 1F).

### Relationship between internalized HIV stigma and fear of progression in PLWHIV

**Fig. 2:**
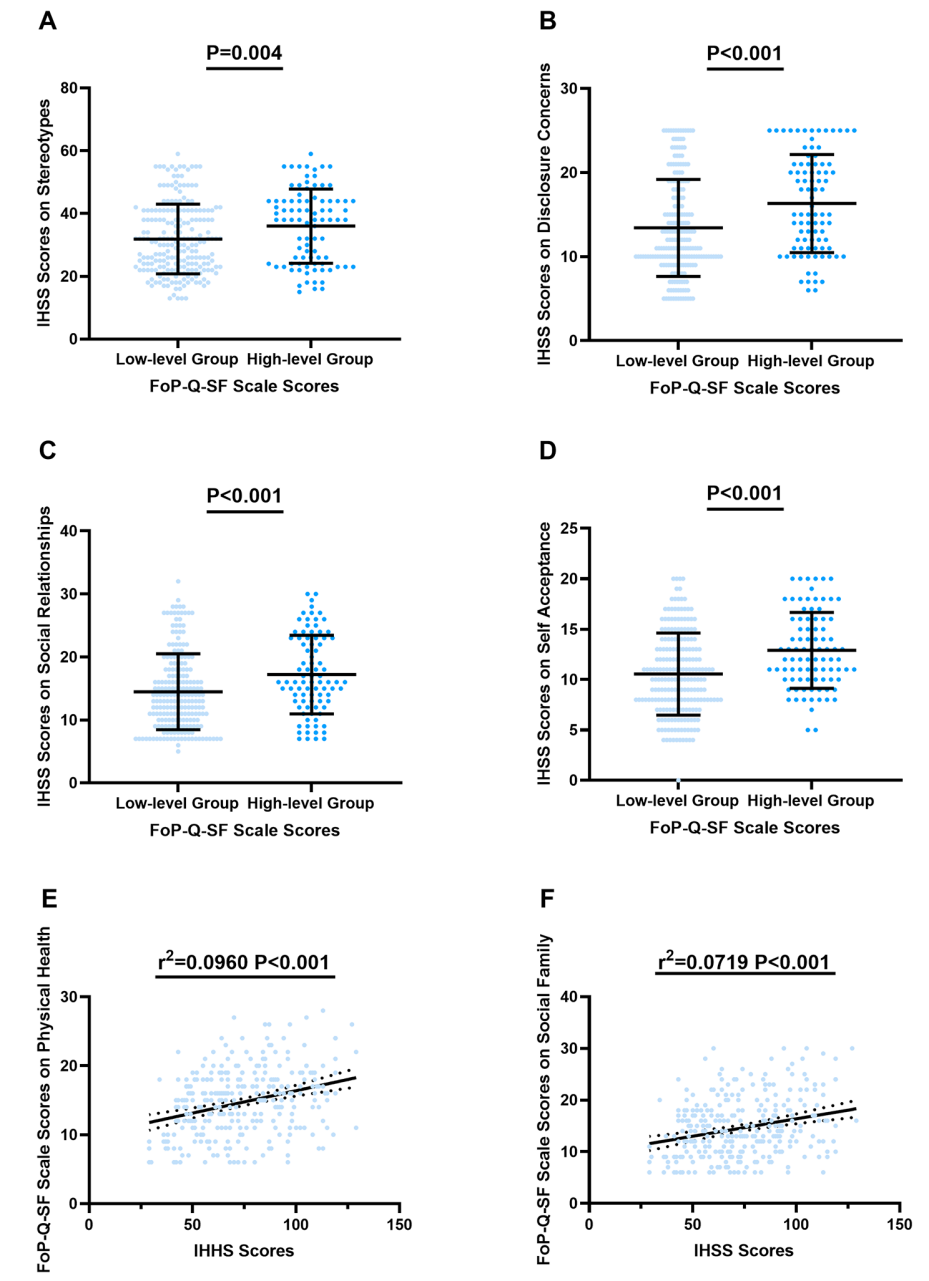
Relationship between FoP and IHSS The comparison of IHSS scores pertaining to stereotypes **(A)**, disclosure concerns **(B)**, social relationships **(C)** and self-acceptance **(D)** between groups characterized by low-level and high-level FoP. **(E)** The relationship between FoP-Q-SF scale scores on physical health and IHSS scores among the PLWHIV. **(F)** The relationship between FoP-Q-SF scores on social family and IHSS scores among the PLWHIV. Abbreviations: FoP: Fear of Progression; FoP-Q-SF: Fear of Progression Questionnaire-Short Form; IHSS: Internalized HIV Stigma Scale; PLWHIV: People Living with HIV

Then, we explored the relationship between IHS and FoP. Compared with the low-level FoP group, the high-level FoP group was more likely to have higher scores in all dimensions of IHSS: stereotypes (36.01 ± 11.83 versus 31.87 ± 11.11, *P* = 0.004) (Fig. 2A), disclosure concerns (16.32 ± 5.83 versus 13.42 ± 5.77, *P* < 0.001) (Fig. 2B), social relationships (17.60 ± 6.25 versus 14.48 ± 6.02, *P* < 0.001) (Fig. 2C) and self-acceptance (12.90 ± 3.76 versus 10.55 ± 4.08, *P* < 0.001) (Fig. 2D). IHSS scores were positively correlated with FoP-Q-SF scores on physical health (r^2^ = 0.0960, *P* < 0.001) (Fig. 2E) and social family (r^2^ = 0.0719, *P* < 0.001) (Fig. 2F).

### Factors associated with fear of progression among PLWHIV

**Fig. 3:**
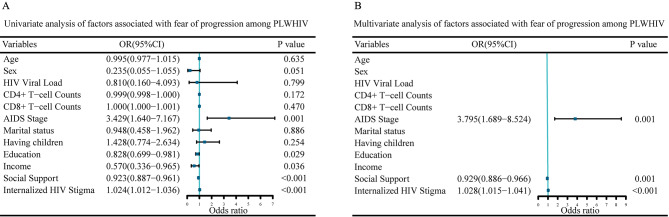
Univariate analysis and multivariate analysis of factors associated with FoP among PLWHIV **(A)** Forest plot of univariate analysis. **(B)** Forest plot of multivariate analysis. Abbreviations: AIDS: Acquired Immunodeficiency Syndrome; FoP: Fear of Progression; PLWHIV: People Living with HIV

Multivariate analysis showed that being at the AIDS stage (OR = 3.795, *P* = 0.001), social support (OR = 0.929, *P* = 0.001) and IHS (OR = 1.028, *P* < 0.001) were independent factors related to FoP level among PLWHIV (Fig. 3).

### Factors associated with medical coping mode among PLWHIV

**Fig. 4:**
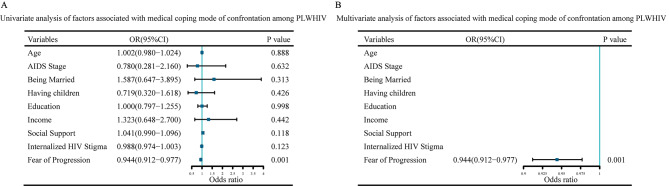
Univariate analysis and multivariate analysis of factors associated with medical coping mode of confrontation among PLWHIV **(A)** Forest plot of univariate analysis. **(B)** Forest plot of multivariate analysis. Abbreviations: AIDS: Acquired Immunodeficiency Syndrome; PLWHIV: People Living with HIV

**Fig. 5:**
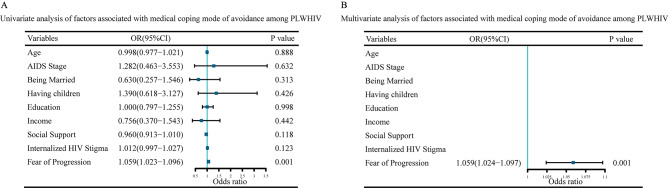
Univariate analysis and multivariate analysis of factors associated with medical coping mode of avoidance among PLWHIV **(A)** Forest plot of univariate analysis. **(B)** Forest plot of multivariate analysis. Abbreviations: AIDS: Acquired Immunodeficiency Syndrome; PLWHIV: People Living with HIV

Then, we assessed the factors associated with confrontation and avoidance coping mode scores among PLWHIV. It’s showed that FoP was the significant factor associated with confrontation scores (OR = 0.944, *P* = 0.001) (Fig. 4) and avoidance scores (OR = 1.059, *P* = 0.001) in multivariate analysis (Fig. 5).

## Discussion

**Fig. 6:**
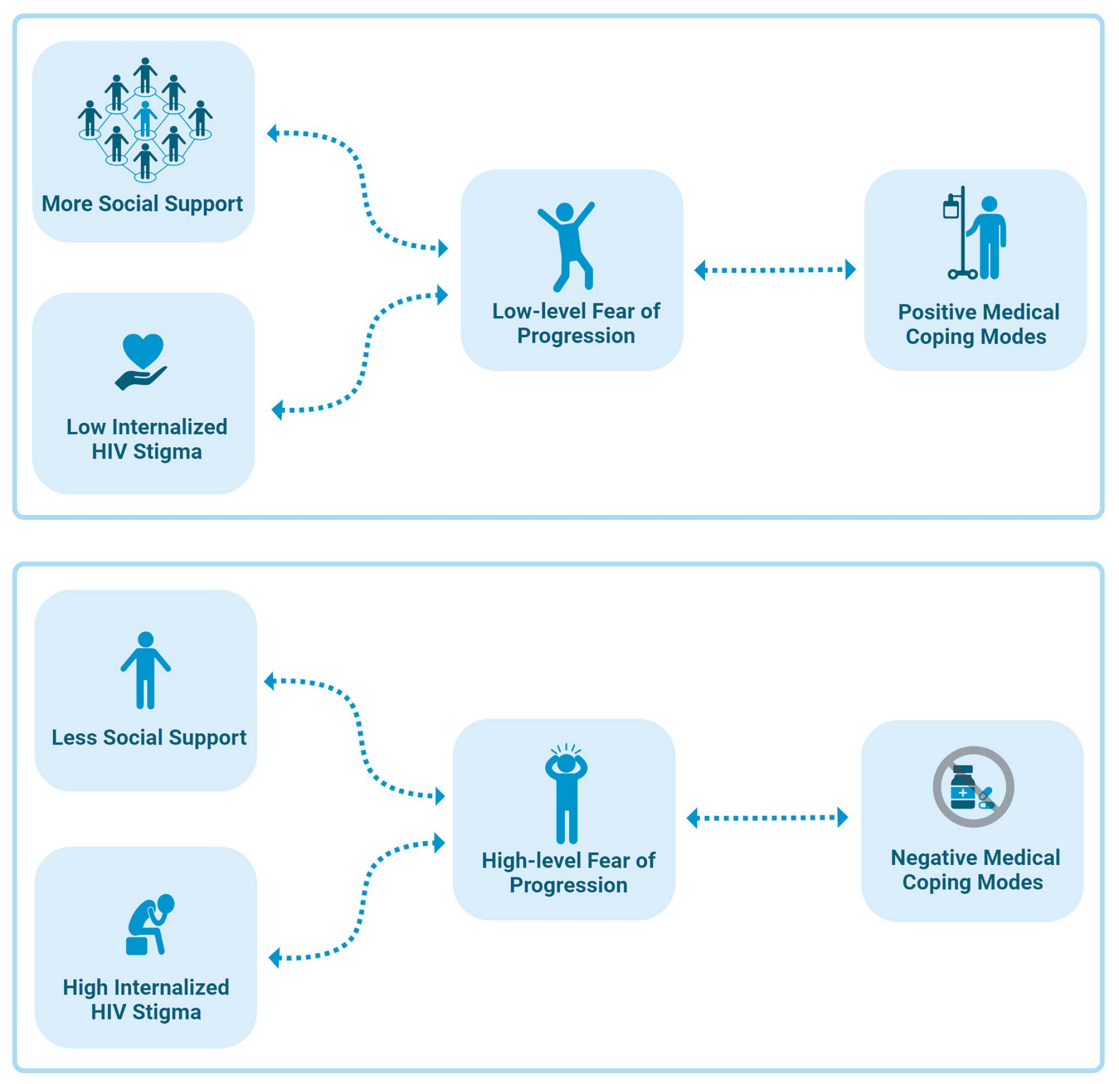
The relationship among the factors including fear of progression, social support, internalized HIV stigma and medical coping modes is summarized. Image elaborated with BioRender

This study is the first to examine the relationship between FoP and MCM in PLWHIV. We found that social support and positive MCM are negatively correlated with FoP, whereas internalized stigma surrounding HIV has a positive correlation with FoP (Fig. 6).

Notably, the FoP-Q-SF was used to assess the FoP level among PLWHIV with good reliability. Of the 303 participants, 88 (29.04%) were found to meet the diagnostic criteria for dysfunctional FoP, suggesting that the psychological impact of FoP among PLWHIV should be given greater attention. The FoP-Q-SF has been widely used to assess FoP in patients, particularly those with cancer and other chronic diseases [8, 27–29]. The prevalence of dysfunctional FoP among cancer patients has been found to range from 5.1% in patients with multiple types of cancer to 68.4% in breast cancer patients [9, 30]. Our data provides additional insights into the landscape of FoP among PLWHIV.

In this study, there were no significant differences in sex, age, being married or having children between the high-level FoP group and the low-level FoP group among PLWHIV. However, FoP has been found to be associated with certain demographic characteristics, including sex, age, marital status, having children, education level, income level, and employment status among cancer patients. Specifically, female patients, younger patients, those who were married, had children, had low education, low income, and were employed tended to have higher levels of FoP [31–38]. This may be due to the fact that the majority of participants in this study were male (295/303, 97.4%) and young adults (with an average age of 31.62 ± 17.82). This is roughly in line with the sex and age distribution of PLWHIV in China [39, 40]. Consistent with previous studies [35–38], the high-level FoP group had a lower education level and lower income level than the low-level FoP group.

In our research, the disease stage was associated with PLWHIV’s FoP level. The high-level FoP group had a higher proportion of participants in the AIDS stage compared to the low-level FoP group, and being at AIDS stage was associated with high FoP. FoP levels vary among patients with different diseases, and even among patients with the same disease, the FoP level can differ depending on the stage and phase of the disease [9, 24]. Treatment regimens can also affect FoP levels [41]. In patients with uveal melanoma, their FoP level decreased as time passed after diagnosis, possibly due to the effectiveness of psycho-oncological interventions [42]. Patient satisfaction with medical staff and communication was found to be negatively correlated with FoP level, while the perception of disease symptoms, such as self-perceived pain and declining physical functioning, was associated with high levels of FoP in cancer patients [43–45]. As we all know, being at the AIDS stage is associated with the development of opportunistic infections and tumours [16–18]. Thus, the increased exposure to these conditions may contribute to the higher levels of FoP observed in this group. These results highlight the importance of considering disease stage when assessing and addressing FoP among PLWHIV.

Finally, in our study, we found that higher scores in objective support, subjective support, and utilization of support were all related to low-level FoP, indicating that increasing social support may be beneficial in reducing FoP in PLWHIV. The abilities and interpersonal relationships of PLWHIV were also associated with FoP. Factors such as self-efficacy, competency in managing distress, resilience and posttraumatic growth were all negatively correlated with FoP [34, 46–48]. These findings indicate that psychological ability might play a role in alleviating FoP. Social support was also an important influencing factor for FoP. Previous studies have shown a significantly negative correlation between social support and FoP in patients with cancer or chronic disease [47, 49–51]. For instance, support and understanding from medical staff have been shown to effectively alleviate the fear of disease progression and recurrence among patients with breast cancer [43]. We also evaluated their IHS level and found that IHSS scores were positively associated with FoP. It is important to note that people with infectious diseases, such as PLWHIV, often face stigma and discrimination, which may contribute to their FoP [52]. These findings suggest that the causes of FoP in PLWHIV may differ from those in patients with other chronic noncommunicable diseases.

In the case of PLWHIV of our research, high FoP was associated with more avoidance, a negative coping mode, but also less confrontation, a positive coping mode. FoP has been identified as a detrimental factor for the QoL of patients across different diseases [29, 32, 45]. It has also been reported as one of the main reasons for patients with prostate cancer to abandon active surveillance [53]. Patients with high-level FoP tend to cope with their illness through distraction and avoidance, as observed in breast cancer patients [54]. Interestingly, in colon cancer patients, FoP was associated with the adoption of healthier lifestyles, such as reducing smoking, alcohol consumption, and unhealthy food intake [55]. In summary, the interplay between FoP and coping strategies is a widely observed phenomenon in various diseases. While moderate levels of FoP can motivate individuals to adopt healthier lifestyles and behaviors that are beneficial for disease management, excessive FoP is correlated wih negative medical coping strategies. Hence, understanding the dynamics between FoP and coping strategies is crucial in designing effective interventions to improve the overall well-being and QoL of patients.

Our research had a few limitations. Firstly, the majority of PLWHIV participants in this study were young adult males, which limits the generalizability of our findings to other populations. Secondly, the classification criteria for distinguishing high and low levels of FoP used in this study were based on criteria established for cancer patients. Although the FoP-Q-SF showed good reliability in our study, further research is needed to validate these criteria in PLWHIV populations. Thirdly, this research was a cross-sectional study with a lack of causality. To address these limitations, future research should include longitudinal cohort studies that evaluate the potential impact of FoP on treatment adherence and QoL.

## Conclusion

Our research underscores that being at AIDS stage is a significant factor associated with high levels of FoP. Notably, dysfunctional FoP is found to correlate with negative medical coping strategies. The outcomes highlight the necessity for early assessment of FoP and psychological interventions in PLWHIV, which needs further prospective study to validate.

## Data Availability

The data that support the findings of this study are available from the corresponding author upon reasonable request.

## Abbreviations

AIDS: Acquired Immunodeficiency Syndrome
FoP: Fear of Progression
FoP-Q-SF: Fear of Progression Questionnaire-Short Form
HAART: Highly Active Antiretroviral Therapy
IHS: Internalized HIV Stigma
IHSS: Internalized HIV Stigma Scale
MCM: Medical Coping Mode
MCMQ: Medical Coping Modes Questionnaire
PLWHIV: People Living with HIV
QoL: Quality of Life
SSRS: Social Support Rating Scale
